# A Five Years Old Child with Failure To Thrive and Vomiting Presenting as a Diagnostic Dilemma: A Case Report

**DOI:** 10.31729/jnma.5134

**Published:** 2020-08-31

**Authors:** Anita Lamichhane, Rupesh Sharma, Ramana Rajkarnikar, Rubee Awale, Prapti Shrestha, Nava Chandra Oli

**Affiliations:** 1 Department of Pediatrics, Lumbini Medical College and Teaching Hospital, Pravas, Palpa, Nepal; 2 Department of Radiology, Lumbini Medical College and Teaching Hospital, Pravas, Palpa, Nepal; 3 Department of Pediatric Surgery, Kanti Children's Hospital, Kathmandu, Nepal

**Keywords:** *exploratory laparotomy*, *failure to thrive*, *intestinal malrotation*

## Abstract

Vomiting with failure to thrive in older children is a diagnostic challenge due to the diversity in the diagnosis. We report a case of a five-years-old girl with failure to thrive, history of recurrent vomiting and intermittent colicky pain abdomen since 45 days of life. Intestinal malrotation with Ladd's band was diagnosed based on clinical acumen, high-resolution computed tomography, barium follow through and intraoperative findings. Exploratory laparotomy with Ladd's procedure was performed under general anesthesia which showed malrotation at the duodenojejunal junction with a short route of mesentery with floating caecum with Ladd's band. Failure to thrive with malrotation of the gut in the older age group is rare in itself. As there are very few cases reported in this age group, so we undertook to report this case to increase the awareness of knowledge concerning the diagnosis and timely management to prevent the comorbidity of this condition.

## INTRODUCTION

Intestinal malrotation is a congenital position of the duodenojejunal junction, a potentially life-threatening complication. There is an increased risk of bowel obstruction with necrosis and acute or chronic volvulus; involving both the small and large intestine. About 75-85% of these patients are diagnosed in early infancy whereas in the rest, the diagnosis can be delayed to childhood or even adulthood.^1^ The real incidence of malrotation is not possible to estimate as most children do not present with any symptoms throughout their lives.^2^

As vomiting with failure to thrive in older children is a diagnostic challenge, so we report a case of a five-years-old girl with failure to thrive, history of recurrent vomiting and intermittent colicky pain abdomen.

## CASE REPORT

A five years old girl child presented in the emergency department with multiple episodes of vomiting since one and half months of age. Vomitus was non-bilious and not mixed with blood, six to seven episodes per day, and projectile in nature. It had increased from the last few days. There was associated periumbilical pain which was intermittent in nature, colicky, non-radiating, and relieved with vomiting. She had normal appetite despite the abdominal pain. Later on, vomiting became bile stained. There was no history of fever, cough, yellowish discoloration of eyes and body, headache, seizure, blurring of vision, no contact with tuberculosis patients, no history of any surgical procedure done. She had normal bladder and bowel habits. She was not gaining weight.

She was born full-term by normal vaginal delivery, birth weight 2.5 kg, urine and the stool were passed in the first 24 hours of life postnatally. The neonatal period was uneventful. There was no gross abnormality or any congenital birth defect in the baby. Antenatal history was unremarkable. She started having intermittent vomiting since 45 days of life for which she was treated at various local pharmacies and hospitals. After 3-4 months of vomiting free period, she always used to land in the emergency department with vomiting and pain abdomen which was self-limiting but recurrent.

At the time of presentation in the emergency department, the child was conscious and alert, well oriented, comfortably lying on the bed. She was thin built and wasted with no dysmorphic facies. Her weight was 12 kg, height was 101 cm, Body mass index (BMI) was 12.7 kg/m^2^ (-2 to -3 z score). She was pale and mild dehydration was present. There was no jaundice, edema, cyanosis, and lymphadenopathy. Other vital signs were within normal limits. The cardiovascular and respiratory examination were within normal limits. Arterial blood gases showed hyperchloremic metabolic alkalosis. There was mild hypokalaemia while other electrolytes were within normal limits. Total leucocyte count was normal, renal function and liver function tests were within normal limits' C-reactive protein was not raised. Per abdominal examination revealed a non-tender distended epigastric region. There was no flank fullness, no organomegaly, bowel sounds were hyperactive on auscultation.

**Figure 1.**
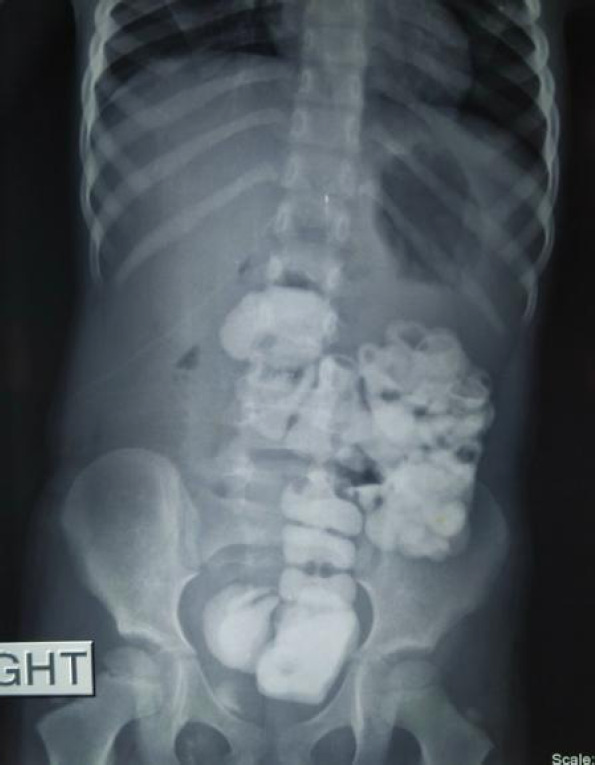
Barium follow through shows left sided large bowel loops.

Keeping in mind the history, physical examination, and laboratory values, we had a suspicion of incomplete pyloric stenosis. So initially ultrasonography (USG) abdomen was done which showed whirling of vessels surrounding-bowel loops. This was in contrast with our working diagnosis. So, we opted for barium meal follow-through (Figure 1).

**Figure 2.**
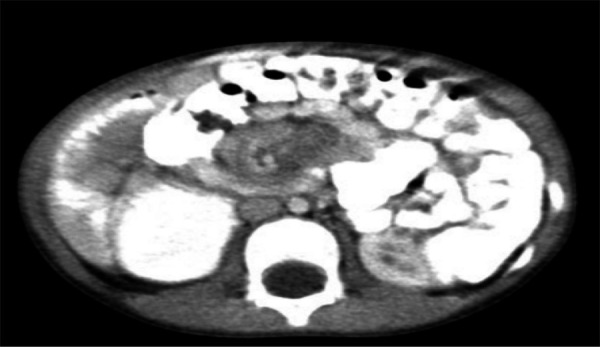
CECT shows predominantly left sided large bowel loops with whirlpool sign and inverted SMA/SMV relation.

This arouse suspicion for other possible diagnosis. Hence, contrast-enhanced computed tomography (CECT) of the abdomen was done. CECT showed the small bowel loops predominantly located in the right side and the large bowel loops in the left side of the peritoneal cavity. There was evidence of a segment of small bowel loop in the region of proximal jejunum noted to rotate around the mesentery giving whirlpool appearance. The superior mesenteric artery was noted to lie in the right side of superior mesenteric vein (representing inverted SMA/SMV relation). The duodeno-jejunal junction did not cross the midline. Mild ascites with few enlarged mesenteric nodes and no evidence of pneumoperitoneum or abnormal air-fluid level was noted. The findings were consistent with intestinal malrotation with midgut volvulus (Figure 2).

**Figure 3.**
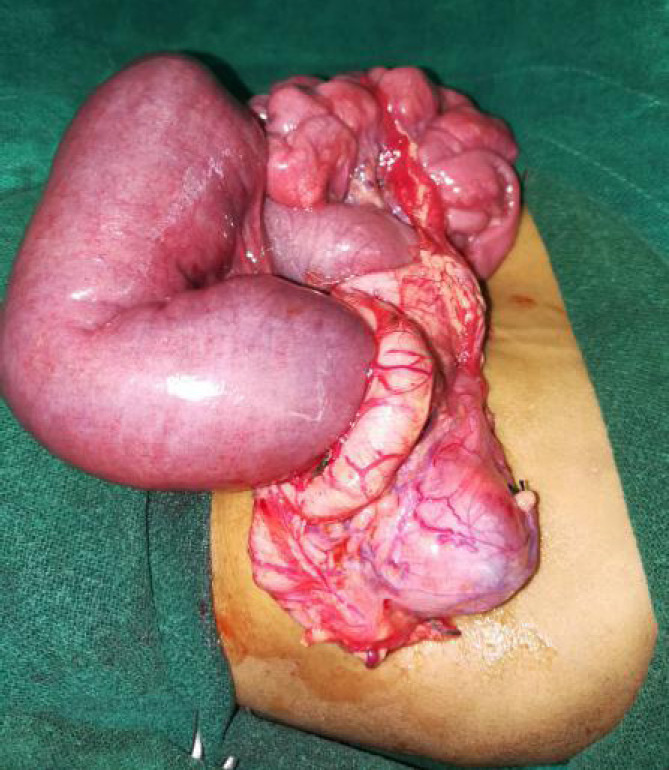
Hugely dilated proximal jejunum with malroated mesentery and distal jejunum and ileum with floating caecum.

**Figure 4.**
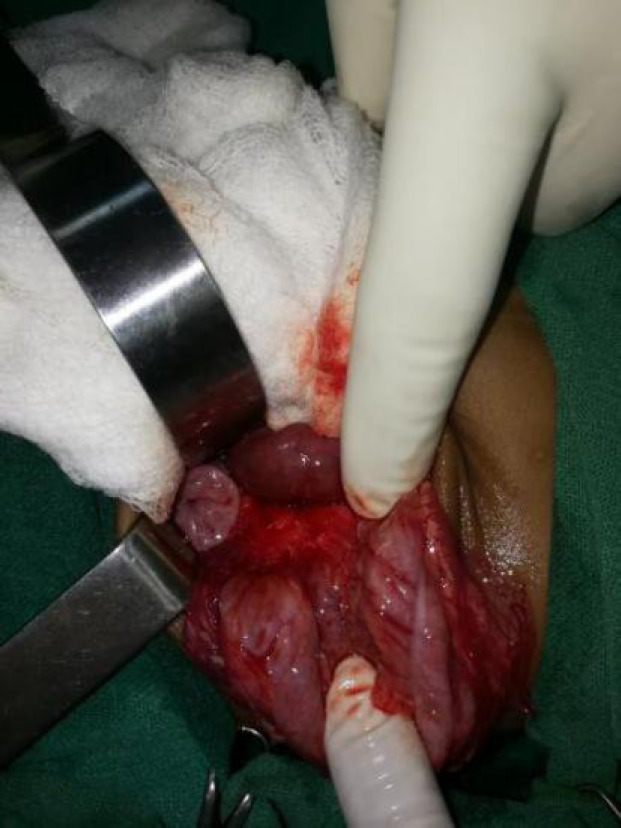
After derotation of gut and decompression of proximal gut, showing superior mesenteric vessels.

She was managed conservatively initially with intravenous fluids and was stabilized first. As there was no Pediatric surgeon available in our institute, the child was referred to Kanti Children's Hospital, Kathmandu, Nepal for further consultation and management. She underwent exploratory laparotomy which revealed malrotation of the gut at the duodenojejunal junction with short mesentery and floating caecum (Figure 3), (Figure 4) with Ladd's band. Ladd's procedure along with appendicectomy was done.

Post-operatively patient was treated with IV fluids and IV antibiotics with daily dressing of the operated site. She was discharged on the seventh postoperative day after removing the sutures. The hospital stay period was uneventful. Then she was assessed again after seven days followed at three weeks. There was no wound infection and at three weeks follow up, scar was healthy. There was no postprandial vomiting or nausea, abdominal pain. The child was gaining weight slowly at three weeks of discharge from the hospital.

## DISCUSSION

Malrotation of the gut is due to complete or partial failure of 270 degree of clockwise rotation of the midgut around the superior mesenteric pedicle; it results in the anomalous position of small bowel loops in the right side with absence of ligament of Treitz and appendix, caecum and ascending colon on the left side.^3^ Malrotation is commonly diagnosed in infancy with a 55% incidence of a clinical emergency related to malrotation in the first week of life and an 80% incidence within the first month of life.^4^ A peritoneal fibrous band - also known as Ladd's band can compress duodenum causing the duodenal obstruction. Intestinal malrotation is a disease of the newborn as it frequently manifests in the first month of life; adult manifestation is very rare.^5^ However, older children and adolescents are likely to present with recurrent abdominal pain, intermittent obstructive symptoms, or failure to thrive due to intestinal obstruction or intestinal ischemia.^6,7^ Beyond the neonatal period, patients with intestinal malrotation tend to present with chronic and non-specific symptoms, and therefore these patients are commonly associated with delayed diagnosis.

Approximately 85% of malrotation cases present in the first two weeks of life.^8^ In our case, the child presented with failure to thrive and vomiting with intermittent abdominal pain. These signs are the typical presentation of typical chronic midgut volvulus.^9^ In general, symptomatic patients with malrotation should be treated with surgical intervention. The classic treatment for incomplete intestinal rotation is the Ladd procedure, which requires mobilization of the right colon and cecum by the division of Ladd bands, mobilization of the duodenum, division of adhesions around the superior mesenteric artery to broaden the mesenteric base, and an appendectomy.^10^

The Ladd's procedure is the standard surgical management of midgut volvulus and intestinal malrotation. Most evidence on the outcomes of the Ladd's procedure originates from studies on the Pediatric population and the recurrence among children who have had a Ladd's procedure is low (2-7%). Malrotation of gut without volvulus beyond infancy may go unnoticed and undiagnosed due to its non-specific signs and symptoms. The surgical management of the midgut volvulus consists of Ladd's procedure. Timely diagnosis and management improves the quality of life of these children.
